# Characterization of a Group of MITEs with Unusual Features from Two Coral Genomes

**DOI:** 10.1371/journal.pone.0010700

**Published:** 2010-05-18

**Authors:** Shi Wang, Lingling Zhang, Eli Meyer, Mikhail V. Matz

**Affiliations:** 1 Section of Integrative Biology, University of Texas at Austin, Austin, Texas, United States of America; 2 Waggoner Center for Alcohol and Addiction Research, University of Texas at Austin, Austin, Texas, United States of America; University of Edinburgh, United Kingdom

## Abstract

**Background:**

Miniature inverted-repeat transposable elements (MITEs), which are common in eukaryotic genomes, are small non-coding elements that transpose by utilizing transposases encoded by autonomous transposons. Recent genome-wide analyses and cross-mobilization assays have greatly improved our knowledge on MITE proliferation, however, specific mechanisms for the origin and evolution of MITEs are still unclear.

**Principal Findings:**

A group of coral MITEs called *CMITE* were identified from two corals, *Acropora millepora* and *Acropora palmata*. *CMITEs* conform to many common characteristics of MITEs, but also present several unusual features. The most unusual feature of *CMITEs* is conservation of the internal region, which is more conserved between MITE families than the TIRs. The origin of this internal region remains unknown, although we found one *CMITE* family that seems to be derived from a *piggyBac*-like transposon in *A. millepora*. *CMITEs* can form tandem arrays, suggesting an unconventional way for MITEs to increase copy numbers. We also describe a case in which a novel transposable element was created by a *CMITE* insertion event.

**Conclusions:**

To our knowledge, this is the first report of identification of MITEs from coral genomes. Proliferation of *CMITEs* seems to be related to the transposition machinery of *piggyBac*-like autonomous transposons. The highly conserved internal region of *CMITEs* suggests a potential role for this region in their successful transposition. However, the origin of these unusual features in *CMITEs* remains unclear, and thus represents an intriguing topic for future investigations.

## Introduction

Transposable elements (TEs) are prevalent in the genomes of all animals and plants, and are often thought of as selfish or parasitic elements [1]. The relationship between TEs and their hosts has been described as an arms race, with the TEs trying to increase their copy number in the host genome and the host trying to protect the integrity of its genetic content [2]. This arms race can lead to enhanced genome plasticity and thus drive host genome evolution (for recent reviews, see [2], [3]).

Eukaryotic TEs can be divided into two major classes, retrotransposons (class I) and DNA transposons (class II), on the basis of the presence or absence of RNA as a transposition intermediate [4]. With few exceptions, classic “cut-and-paste” DNA transposons have terminal inverted repeats (TIRs) at both ends and transpose using the so-called “cut-and-paste” mechanism (for a review, see [5]). Some DNA transposons are autonomous, encoding their own transposases, while others are nonautonomous. Nonautonomous DNA transposons maintain transposition activity by retaining the *cis* sequences (e.g. TIRs or in some cases, subterminal repeated sequences) recognized by *trans* transposases from autonomous DNA transposons.

Miniature inverted-repeat transposable elements (MITEs) are a special class of nonautonomous DNA transposons that can transpose by “borrowing” the transposition machinery of autonomous DNA transposons with similar TIR signals [6]–[9]. MITEs have a suite of well known characteristics such as small size (usually less than 500 bp), conserved TIRs, and the absence of protein-coding sequences [10]. In contrast to typical nonautonomous DNA transposons, MITEs are highly homogeneous in size and are usually present in genomes in very high copy numbers. Because MITEs do not encode transposases, their classification is mainly based on shared TIR and target site duplication (TSD) sequences. To date, most MITEs can be classified into seven superfamilies that include Tc1/*mariner* (*Stowaway*-like MITEs), *PIF*/*Harbinger* (*Tourist*-like MITEs), *piggyBac*/TTAA and *hAT* [10]. Although recent genome-wide analyses and cross-mobilization assays have greatly improved our knowledge on MITE proliferation [8], [9], [11]–[14], specific mechanisms for the origin and evolution of MITEs are still unclear.

Here, we present the first report of a group of coral MITEs called *CMITE*, which were identified from whole-genome shotgun (WGS) sequences of two coral species, *Acropora millepora* and *Acropora palmata*. Although *CMITEs* conform to many common characteristics of MITEs, they also present the following unusual features: (i) highly conserved internal region but less conserved TIRs, (ii) formation of tandem arrays, and (iii) *de novo* assembly of a novel TE.

## Materials and Methods

### Sequences

WGS sequences of *A. millepora* and *A. palmata* were downloaded from the National Center for Biotechnology Information (NCBI) database. There were 14625 and 11024 entries for *A. millepora* and *A. palmata*, respectively.

### Bioinformatic analysis of *CMITE* elements

*CMITEs* with matching TIRs (13∼14 bp in length) were first identified using the FINDMITE program [15]. In order to search for possible related elements, a 60 bp consensus sequence (5′-AGGGGTTCCCCATTGACGAGTAAAATCGTCTGGCGTTAGACAGAGTAAAATCTATAAGTG-3′) from the internal conserved region of *CMITEs* was used for blastn search [16]. A cutoff value of e ≤10^−5^ was used as the significance threshold for the comparison.

Multiple sequence alignment was performed using the MegAlign program (part of the DNASTAR software package) and sequence alignments were manually refined. A formula was adopted to estimate the copy number of *CMITEs* in the genome: copy number  =  (number in database × genome size)/database size [15]. This calculation was only possible for *A. millepora*, since there is a previously published estimate of 200 Mbp for this genome size [17].

### Isolation of *piggyBac*-like transposons from *A. millepora* genome

Using *piggyBac*-like elements from RepBase 13.05 (n = 73, [18]) as queries (tblastx, e≤10^−4^), we identified 14 distinct *A. millepora piggyBac*-like sequences. Eleven of them came from the *A. millepora* larval transcriptome (NCBI ID: SRA003728, [19]), and 3 came from the WGS sequences.

Two approaches were used to isolate *piggyBac*-like transposons from *A. millepora* genome. In the first approach (direct PCR), polymerase chain reaction (PCR) primers were designed based on the TIR sequences of *CMITE* family I, II and III in an effort to isolate MITE family-specific *piggyBac*-like transposons. PCR amplifications were set up in a 20 µL volume composed of 10 ng *A. millepora* genomic DNA, 0.5 µM each primer, 0.2 mM dNTP, 1× Phusion HF buffer and 0.4 U Phusion hot start high-fidelity DNA polymerase (NEB, Ipswich, MA) in a DNA Engine Tetrad 2 thermal cycler (Bio-Rad, Hercules, CA). All cycling began with an initial denaturation at 98°C for 30 s, followed by 35 cycles of 98°C for 10 s, 60°C for 30 s, 72°C for 5 min, and a final extension at 72°C for 10 min. PCR products were detected by agarose gel electrophoresis. PCR product containing fragments in the desired size range (i.e. 2–6 kb) was purified using QIAquick PCR purification kit (Qiagen, Valencia, CA). Because Phusion DNA polymerase generates blunt-end PCR products, 3′ A overhangs must be added to the blunt PCR product before TA cloning. The A-addition reaction was set up in a 10 µL volume composed of ∼200 ng purified PCR product, 0.2 mM dATP, 1× ThermoPol buffer and 1 U *Taq* DNA polymerase (NEB, Ipswich, MA), and incubated at 72°C for 30 min. After treatment, PCR products were ligated into pGEM-T vector (Promega, Madison, WI) and subsequently transformed into TOP10 competent *Escherichia coli* cells (Invitrogen, Carlsbad, CA). Recombinant clones were screened for inserts of correct size, and then were sequenced at the DNA Core Facility at UT Austin. In this approach, the exact TIR sequences of a *piggyBac*-like element remain unknown since the TIR region of this element serves as a primer-binding site. An adaptor-ligation PCR method [20] was utilized to obtain the TIR sequences of a given *piggyBac*-like element. To prepare the adaptor-ligated DNA, 200 ng of *A. millepora* genomic DNA was digested with 5 U *Mse*I (NEB, Ipswich, MA) at 37°C for 3 h. The reaction was inactivated at 65°C for 20 min. A ligation solution containing 50 pMol *Mse*I-adapter (5′ CAGCAGACTTGAGGTCGTGGTGCTGAGTGCAGTG 3′ and 5′ TACACTGCACTCAGC-NH_2_ 3′), 200 U T4 DNA ligase (NEB, Ipswich, MA) and 1 mM ATP (NEB, Ipswich, MA) was added, and the resultant solution was incubated at 16°C for 16 h. PCR amplifications were set up in a 20 µL volume composed of 10 ng adaptor-ligated DNA, 0.1 µM adaptor-specific primer (5′ GCCTTGCCAGCCCGCTTGTCAGCAGACTTGAGGTCGTGGT 3′), 0.1 µM transposon-specific upstream or downstream primer, 0.2 mM dNTP, 1× Advantage 2 PCR buffer and 1× Advantage 2 Polymerase Mix (Clontech, Mountain View, CA). All cycling began with an initial denaturation at 94°C for 5 min, followed by 35 cycles of 94°C for 30 s, 60°C for 30 s, 68°C for 30 s, and a final extension at 68°C for 10 min. PCR products were then cloned and sequenced as described above.

In the second approach, inverse PCR was utilized in an effort to isolate full *piggyBac*-like transposons based on the 14 *A. millepora piggyBac*-like sequences. A 600-ng aliquot of *A. millepora* genomic DNA was digested with 5 U *Nco*I, *Bgl*II and *Bam*HI (NEB, Ipswich, MA) respectively at 37°C for 3 h. Digested DNA was purified using QIAquick PCR purification kit (Qiagen, Valencia, CA), and was self-circularized in a final volume of 300 µL using T4 DNA liagse (NEB, Ipswich, MA) at 16°C for 16 h. After purification, ∼10 ng of ligated DNA was used for PCR amplification. PCR amplification, TA cloning and sequencing were followed the same procedure in the direct PCR approach. Primers used in the two approaches were designed based on several principles as described by Matz [21] so that all PCR amplifications could be achieved at the same annealing temperature.

### Phylogenetic analysis of *A. millepora piggyBac*-like transposons

Transposase protein sequences were aligned using the ClustalW method [22]. The protein sequence alignment is available in the Supplementary Dataset S1. Phylogenetic analysis was performed with the program MrBayes 3.1 [23]. The appropriate model of evolution was identified as WAG+G+I [24] using the MCMC model-jumping method. The MCMC chain was run for 1,000,000 generations with a sample frequency of 200. In total, 5000 trees were produced, of which the first 4500 were discarded as burn-in while summarizing the data.

## Results

### Discovery and characterization of *CMITE* families

**Figure 1:**
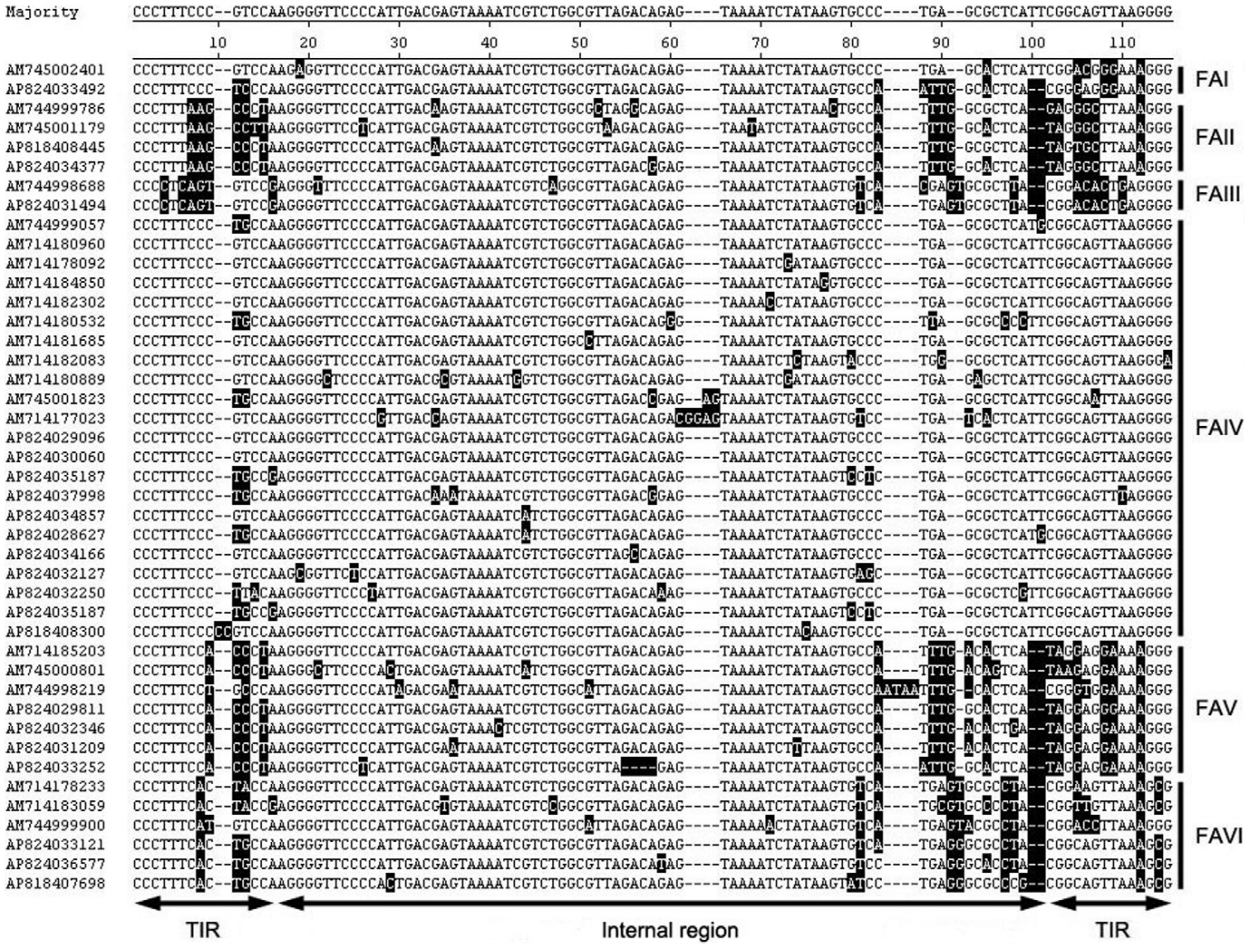
Sequence alignment of *CMITE* elements from two coral species. *CMITE* elements are named with initial capitals of species name (AM: *Acropora millepora*; AP: *Acropora palmata*) followed by an accession number. Consensus sequence is shown at the top of alignment. The terminal inverted repeat (TIR) and the conserved internal region are indicated by double arrows. Bases different from consensus sequence are shaded. FAI to FAVI represents family I to family VI.

When searching for MITEs in the WGS sequences of *A. millepora*, our attention was quickly turned to several predicted MITEs (which we later called *CMITE*), which had different TIRs but shared highly conserved sequences in their internal region. Using the FINDMITE program [15], eight *CMITE* elements with matching TIRs (13∼14 bp in length) were initially identified in the WGS sequences of *A. millepora* and *A. palmata*. These *CMITEs* showed many of the characteristic features of MITEs. They were small (about 100 bp) and homogeneous in size. They had TIRs and were flanked by TTAA TSDs. In contrast to most other MITEs, however, the 75-base-long internal region of *CMITEs* was remarkably well conserved across *CMITE* families (Fig. 1). Based on the similarity of their TIRs, eight *CMITE* elements can be classified into three families (family I, II and III) (Table 1), which is also correlated with the variations in their internal regions, except for one case: *AP824033492* had a family II-like internal region.

**Table 1:** Characteristics of *CMITE* families in two coral species.

Family	Consensus TIR^1^ (5′ to 3′)	No. in AM^2^ WGS database	No. in AP^2^ WGS database	Length (bp)	Average sequence identity (%)
I	Left: CCCTTTCCC(G/T)(T/C)CC	1 (18^3^)	1	102/103	89.6
	Right: GG(A/G)(C/A)GGGAAAGGG				
II	Left: CCCTTTAAGCCCTA	2 (36)	2	102	94.5
	Right: TAGGGCTTAAAGGG				
III	Left: CCCCTCAGTGTCCG	1 (18)	1	103	97.4
	Right: CGGACACTGAGGGG				
IV	Left:CCCTTTCCCGTCCA	41 (737)	33	96–107	91.5
	Right:CGGCAGTTAAGGGG				
V	Left:CCCTTTCCACCCTA	6 (108)	14	98–107	87.2
	Right:TGGGAGGAAAAGGG				
VI	Left:CCCTTTCACTGCCA	6 (108)	13	101–108	84.2
	Right:CGGCAGTTAAAGCG				

1 TIR, terminal inverted repeat. Note, family IV, V, and VI seem to have shorter TIRs than other families, but here we show the terminal 14-bp sequences at both ends;

2 AM and AP are initial capitals of species names, *Acropora millepora* and *Acropora palmata*, respectively;

3 expected copy number in the genome, see section *Materials and Methods* for the calculation method.

To identify possible related elements, we used a 60 bp consensus sequence from the most invariant part of the internal region as a query in blastn search against the WGS sequences of *A. millepora* and *A. palmata*. This search identified 88 significant hits from 78 different *A. millepora* WGS entries, and 111 from 94 different *A. palmata* WGS entries. Multiple matches were found in 7 and 12 WGS entries of *A. millepora* and *A. palmata*, respectively. These searches identified an additional 56 full copies of *CMITEs* from *A. millepora*, and 73 from *A. palmata*. Sequence analysis revealed that in comparison to *CMITEs* from families I, II, and III, all these elements had “shorter” TIRs in which the outermost regions matched their “partners” more closely than the innermost regions (Fig. 1). Since the FINDMITE program was mainly designed to identify MITEs with long and matching TIRs, this explained why most *CMITEs* had not been initially identified by that program. Based on the similarity of their TIRs, 113 of these elements (53 from *A. millepora* and 60 *A. palmata*) were classified into three additional families: families IV, V and VI (Table 1). Two of the remaining copies appeared to be degenerated copies of family III elements, and the other 14 were too degenerated to be unambiguously assigned to one of these families. Family IV was the largest of these families, outnumbering the others by a factor of two in *A. palmata* and almost by a factor of seven in *A. millepora* (Table 1). Within each family, there was no characteristic sequence difference between *A. millepora* and *A. palmata* elements, indicating that these families diverged prior to the coral species separation. Based on their observed frequency in *A. millepora* WGS sequences and the estimated genome size, we estimate the total number of *CMITEs* in the *A. millepora* genome at about 1600 copies. To further check for possible related elements in other species, we used the same query sequence to blast against the NCBI nr database, the Repbase database [18], and the WGS database for another coral species, *Porites lobata*. However, only one significant hit was found in the nr database, a partial lactate dehydrogenase (LDH)-like gene sequence (GenBank ID: EU814629) from *A. millepora*. A full copy of the *CMITE* element was located in the presumed intron region of this gene (data not shown).

**Figure 2:**
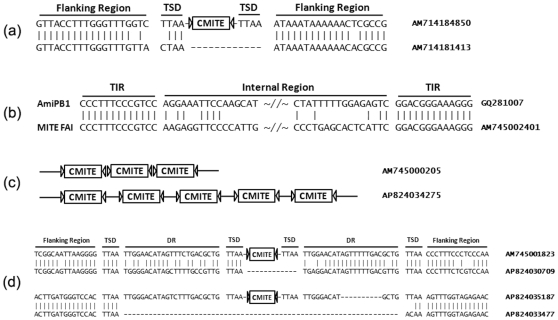
Past mobility, similarity to a *piggyBac*-like transposon, and other features of *CMITEs*. (a) Evidence of past mobility of a *CMITE* element. TSD indicates target site duplication. TIR is represented by a triangle. (b) Sequence comparison between a member of *CMITE* family I and a *piggyBac*-like transposon, *AmiPB1*. Only partial sequences in the internal region adjacent to TIRs are shown. (c) Two representative tandem *CMITE* arrays. (d) Assembly of a *CMITE-IN* element by *CMITE* insertion (top) and evidence of an excision of a *CMITE-IN* element (bottom). DR indicates direct repeat.

An indication of a past transposition event of a *CMITE* was observed among *A. millepora* sequences, where we found two alleles of the same locus, one without a *CMITE* and another with the *CMITE* including the characteristic TSD, TTAA (Fig. 2a).

### Relationship of *CMITEs* and *piggyBac*-like transposons

One of the characteristics of *CMITEs* is the TTAA target site duplication (TSD). To date, only one MITE superfamily, *piggyBac*/TTAA was known to be able to generate TTAA TSDs [10]. This MITE superfamily was supposed to be dependent upon a superfamily of DNA transposon called *piggyBac*. Elements in the *piggyBac* superfamily generally have 12–19 bp TIRs containing a “CC[C/T]T” terminal motif, and generate TTAA TSDs [5]. All the *CMITEs* we initially identified were consistent with these hallmarks of *piggyBac* transposons. When searched against the RepBase database [18], *piggyBac*-like sequences were also found in the WGS databases of *A. millepora* and *A. palmata*, and a recently released *A. millepora* larval transcriptome [19].

In order to investigate the relationship between *CMITEs* and *piggyBac*-like transposons, we decided to isolate *piggyBac*-like transposons from the *A. millepora* genome. Through direct and inverse PCR approaches, six *piggyBac*-like elements were isolated from the *A. millepora* genome (Table 2), and full-length sequences were obtained for four of them. All full-length *piggyBac*-like elements contained the hallmarks of typical *piggyBac* transposons. Partial sequences were obtained for the rest, of which one element has one TIR and a complete open reading frame (ORF), and another one has a complete ORF. Five of these were found in the *A. millepora* larval transcriptome, the expression of these elements during development strongly suggests the presence of functional *piggyBac*-like elements in the *A. millepora* genome.

**Table 2:** Summary of six *PiggyBac*-like transposons in *Acropora millepora*.

Name	TIR^1^ (5′-3′)	ORF^4^ length (aa)	Total length (bp)	Presence in transcriptome	Primers (5′-3′) used in direct or inverse PCR	GenBank ID
*AmiPB1*	Left: CCCTTTCCCGTCC	601	3668	Yes	TIRp^7^: GACTTAACCCTTTCCCGTCC	GQ281007
	Right: GGACGGGAAAGGG				TIRup^8^: CATTGCTCCTATTTTTGGAGAGT	
					TIRdw^8^: CCAAAAAAATGCTTGGAATTTCCT	
*AmiPB2*	Left: CCCTTTA**AC**G**C**CC 2	543	2352	Yes	F: TTGAACTTGACAAGTCCTTCGT	GQ281008
	Right: GG**T**C**CA**TAAAGGG				R: ATATGCCCATGAAGCCCATCA	
*AmiPB3*	Left: CCCTTTCCCTACTA	569	2331	Yes	F: TAAAACCTATCATCCCTTCATCT	GQ281009
	Right: TAGTAGGGAAAGGG				R: ATATGCCCGCAAAACCGACTA	
*AmiPB4*	Left: CCCATTCCCTGCCACA	572^5^	2179	No	F: CCACAAAAGTAATTCCTCGTCAA	GQ281010
	Right: TGTGGCAGGGAATGGG				R: CAAGCAGTACATCGCACTAGA	
*AmiPB5*	Left: CCCTTAGAGACCTA	602	N/A	Yes	F: GAACTTTATCAGGGTCTCATCA	GQ281011
	Right: N/A^3^				R: CCGTCAGTTCATCCCCATCA	
*AmiPB6*	Left: N/A	554^6^	N/A	Yes	F: ATAGGCATGTATTGTTTGAGGTA	GQ281012
	Right: N/A				R: GCCCATCAAGCGTGGGATCA	

1 Terminal inverted repeat;

2 Non-matched bases in TIRs are indicated in bold;

3 Not available;

4 Open reading frame;

5 there are two internal stop codons in this ORF;

6 there is a -1 frameshift in this ORF;

7 TIRp are designed based on the TIR sequences of *CMITE* family I, and 3 irrelevant bases are added to 5′ end of this primer to elevate the melting temperature;

8 TIRup and TIRdw are used to amplify the upstream and downstream TIR sequences of *AmiPB1*, respectively.

**Figure 3:**
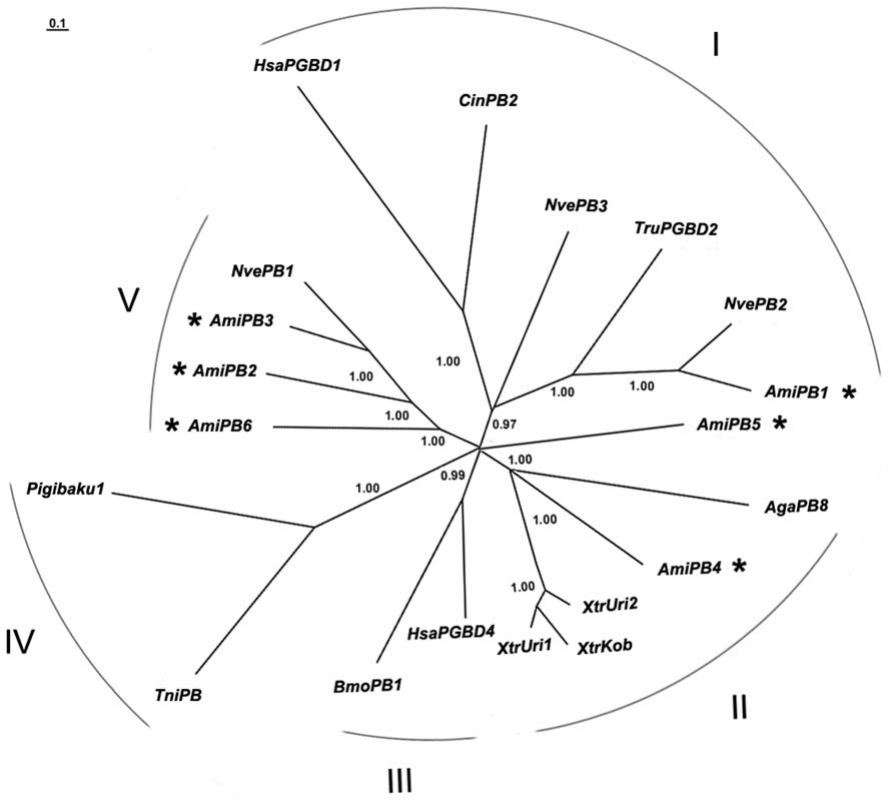
Phylogenetic relationships of *AmiPB1-6* and other elements from the *piggyBac* superfamily based on a Bayesian analysis of transposase protein sequences. *A. millepora piggyBac*-like elements are indicated with asterisks. The edges with posterior probability less than 0.95 are collapsed. Major clades (I-V) are denoted. Note, clade I, II, III and IV correspond to previously identified clades [25]. Species name abbreviations: Ami, *Acropora millepora*; Aga, *Anopheles gambiae*; Bmo, *Bombyx mori*; Cin, *Ciona intestinalis*; Has, *Homo sapiens*; Nve, *Nematostella vectensis*; Thi, *Trichoplusia ni*; Tru (including *pigibaku*), *Takifugu rubripes*; Xtr, *Xenopus tropicalis*. The sequences of *NvePB1-3* are derived from Repbase [18] under the name *piggyBac-1_NV*, *piggyBac-2_NV* and *piggyBac-3_NV*, respectively. Other sequences are either derived from [25] or [38].

Phylogenetic analysis of *AmiPB1-6* and other *piggyBac*-like elements revealed five major clades (Fig. 3). Clades I, II, III and IV correspond to previously identified clades [25]. Clade V is a new clade identified in this study. *AmiPB1 to 6* are grouped in clade I, II and V, which suggests diverse origins of *A. millepora piggyBac*-like elements. Unexpectedly, *AmiPB3* is grouped with *NvePB1* from the sea anemone *Nematostella vectensis* rather than with other *A. millepora* elements in the same clade (Fig. 3, clade V). This may suggest that *piggyBac* clades diverged before the separation of the corresponding Cnidarian orders, Scleractinia and Actiniaria.

*AmiPB1* was the only element isolated through the direct PCR approach. *AmiPB1* and *CMITE* family I share identical TIR sequences, as well as weak sequence similarity in the internal region adjacent to TIRs (Fig. 2b). This suggests that family I is possibly the derivative of *AmiPB1*, and could utilize *AmiPB1* transposase to mobilize in the genome. However, there is no obvious sequence similarity between the most of internal regions of *CMITE* family I and *AmiPB1*. For other *piggyBac*-like elements, except the hallmark terminal TIR motif (i.e., CC[C/T]T), which is necessary for successful transposition of *piggyBac* transposons [26], [27], we did not observe any obvious sequence similarities between these elements and *CMITE* families.

### Tandem *CMITE* arrays

As mentioned above, 7 *A. millepora* and 12 *A. palmata* WGS sequences contained more than one *CMITE* element. Unexpectedly, some of these *CMITE* elements were found in tandem arrays, which typical MITEs usually do not form. Fig. 2c shows two examples of tandem *CMITE* arrays, including one with gaps between the repeated elements. Within these tandem arrays, both the elements themselves and the sequences between them are highly similar, implying that array formation was probably driven by a replication slippage mechanism rather than by independent transposition.

### Identification of the *CMITE-IN* family

We also identified a *CMITE*-related family of elements (which we named *CMITE-IN*) in both *A. millepora* and *A. palmata* WGS sequences. *CMITE-IN* element contains a full copy of the *CMITE* element flanked by direct repeats, and has TTAA at both ends (examples: AM745001823 and AP824035187 in Fig. 2d). Four *CMITE-IN* elements were identified in the *A. millepora* WGS sequences, and one in the *A. palmata* WGS sequences. We estimate there are ∼70 *CMITE-IN* copies in the *A. millepora* genome. A likely prototype of the *CMITE-IN* element was found in an *A. palmata* sequence (*AP824030709*, Fig. 2d), which contains two 23 bp direct repeats and has TTAA at both ends. The two direct repeats are separated by TTAA, which served as a target site for insertion of a *CMITE* element in the genome of another coral (Fig. 2d, top). The *CMITE-IN* element also seems to be a mobile element: we identified a pair of alleles from *A. palmata*, with and without *CMITE-IN* element, which suggest that the *CMITE-IN* can be excised at the position of its protoelement-derived TTAAs (Fig. 2d, bottom). We infer that the allele without the element (*AP824033477*) is a result of the past excision because it retains a possible TSD (Fig. 2d). Although *piggyBac* transposases usually perform precise excision without leaving “footprints” at the target sites [27], [28], imprecise excision events leaving TTAA TSDs in the target site were also observed [29]. In the case presented here, one of these TSDs has apparently mutated into ACAA, possibly as a result of imperfect repair of the double-strand break after transposon excision.

## Discussion

To our knowledge, this is the first report of identification of MITEs from coral genomes. The *CMITEs* described here appear to have originated from *piggyBac-*like transposons. However, in comparison to other MITEs of the same origin [30]–[32], *CMITEs* have the following noteworthy features:

### (i) Highly conserved internal region but less conserved TIRs

The most unusual feature of *CMITEs* is conservation of the internal region, which is more conserved between MITE families than the TIRs. Typically, internal regions of different MITE families are much more dissimilar in size and sequence [10]. In part, the conservation of the internal region in *CMITEs* may result from ascertainment bias, since the internal region was used as query to search for the majority of *CMITEs*. However, there was no such bias while initially detecting *CMITEs* using the FINDMITE program since it was based on TIR similarity only. It is tempting to speculate that the internal region is somehow important for *CMITEs*' transposition. Indeed, a recent study has shown that some internal sequences in MITEs could enhance transposition [9].

We showed that *CMITE* family I seems to be the derivative of a *piggyBac*-like transposon, *AmiPB1*. However, by comparison of *CMITE* family I and *AmiPB1*, we only observed very limited sequence similarity in the internal region adjacent to TIRs (Fig. 2b), and no obvious sequence similarity for the rest of internal region. Thus the origin of internal region of *CMITEs* remains a mystery. Interestingly, a recent study showed that host genomic sequences can be acquired by MITEs and filled in between TIRs through a process called transduplication [14]. This could be a reasonable explanation for the origin of internal region of *CMITEs*. However, the WGS sequences currently available did not include any likely candidates for this putative original sequence, so complete genome sequences (which are unfortunately not yet available for any coral) will likely be required to resolve whether transduplication played a role in these elements.

In contrast to the internal region, TIRs between *CMITE* families are usually less conserved. However, all *CMITE* families preserved the terminal TIR motif (i.e., CC[C/T]T) (Table 1), which is a hallmark of TIRs of *piggyBac* transposons [5], and is necessary for successful transposition of *piggyBac* transposons [26], [27], so it is possible that this TIR motif coupled with the conserved internal region is already sufficient for successful transposition of these *CMITE* families. If this is the case, it might allow for cross-mobilization of these MITEs by various kinds of *piggyBac*-like transposons (Fig. 3), since TIRs of *piggyBac*-like transposons we identified in the *A. millepora* genome also preserved this motif (Table 2).

### (ii) Formation of tandem MITE arrays

Our observations indicate that *CMITEs* can increase their copy numbers not only by transposition, but also by forming tandem arrays. To our knowledge, this is the first report of tandem arrays of full-sized MITEs, although tandem arrays formed by partial internal sequence of a *piggyBac*-MITE have been observed [33]. Specific mechanisms responsible for the *CMITEs* array formation are unclear, but could be related to their similarity to the autonomous *piggyBac* transposons, which are also able to form large tandem arrays [34]. Even if it is the case, however, *CMITEs* seem to be the only *piggyBac*-derived MITEs that retain this ability. This suggests that *CMITEs* contain some unique features that facilitate the formation of tandem arrays.

### (iii) *De novo* assembly of a novel TE

The finding of a novel TE family created by insertion of a *CMITE* suggests an unusual mechanism for the generation of novel TEs. Although we have shown the evidence of past mobility of the *CMITE-IN* element, the transposition mechanism remains unclear. *CMITE-IN* elements are structurally similar to miniature subterminal inverted-repeat transposable element (MSITE), which contain subterminal inverted-repeat (SIR) but no TIRs. Identification of MSITEs has been reported in several studies [35]–[37]. In one particular case, a 7 bp motif in the TIR of *Wuneng* (MITE) was found in the SIR of *Microuli* (MSITE) [37]. Interestingly, both *Wuneng* and *Microuli* can generate TTAA TSDs. Based on these observations, the authors proposed that SIR might play an important role in MSITE transposition by providing key motifs. Since *CMITE-IN* and MSITE share similar TE structure, we speculate that the transposition mechanism of *CMITE-IN* may be also very similar to that of MSITE.

In summary, we present the first report of non-autonomous MITE-like elements (*CMITEs*) from two coral genomes. These elements bear the telltale features of MITEs related to *piggyBac*-like autonomous transposons. We show that the coral genome indeed contains such autonomous transposons, most of which are also transcriptional active, ostensibly providing the transposition machinery for the *CMITEs*. The unusually well-conserved internal region of *CMITEs* suggests a potentially important role in successful transposition. However, the origin of these unusual features in *CMITEs* remains unclear, and represents an intriguing topic for future studies.

## Supporting Information

Dataset S1 AmiPB1-6 and other piggyBac-like transposase protein sequences (fasta and ‘aligned’ formats).
(0.08 MB PDF)
