# Supplementary material for: Characterization of a Group of MITEs with Unusual Features from Two Coral Genomes
Source: PLoS One. 2010 May 18;5(5):e10700. doi: 10.1371/journal.pone.0010700 (PMC2872659; doi:10.1371/journal.pone.0010700)
Supplement: Dataset S1 — AmiPB1-6 and other piggyBac-like transposase protein sequences (fasta and ‘aligned’ formats). (0.08 MB PDF) [file pone.0010700.s001.pdf]

A. fasta format

```
>AmiPB1
----------------------------------------MFSLHYTGPTDTAAFYGAAAARPLPRVPAGPRAPPVVPVLARAGRALGRARGGRAQARGGR------
--------RGRAR--ARGGGQVRGAAVTLHETYDVRDVGNPPLPFNPTRP---IGVHFGQRLLRNAMTTAVEFFNLFFTVELVNNIVTHTNSYAYEHI--
VTHQSYAKSDGSWQEATTDEIRRLIAILIYFGLVRVSDAADNYWSIQSPYLGLWGKCFLSRIRFKALMAMLHVVDPANEPD---------
GDKLRNINPLLEFFKGRCKDLYQPRQNVAVDERIVKSRHR-SGMRQFNKDKPIRWGIKLWVLADSSNGYTIDFNVYIGKVAGQ-------------
DVSANGLGYDVVMKLMN-PYFHQGY--HLYVDNFYTSVTLFKDLFAM--GVGATGTIRECRRDFPENLKDSE-VWAKGKARGSIRWGRDPPCLALQWLDN--
KVVSMLTTIDN-----ANVKKQVTRNMKNPGGGWTNNNVPQPGVIANYNKYMNAVDRSDQILATNNIHR-KCVRWWKTLFFHLID-IAIVNSYILFQEHRANSP---
-DEPALKRPASYSLTNF----REEVVNGLCGF--AEYGPPPPGHVASNLAPPSSEPSQFVTEHIPCFSEEKRTCVVCWQKEKKQYKVKTYCKA-------P------
QCNRYMHITNEKIV------------------------------------
>AmiPB2
-----------------------------------------------------------MASVSDIFDSDSEEEEFFGFSTNDIARADISGE----SDISA------
--------DESFTEDEESDSNSVESGGEEDWSDDLDFVIVEDFNKP-----------TGPTTVLPATATAGDFLNLVFPEDLIQLIVDETNRNAR-----
QKQQQSGTVDKDWMPVNNSDIKAFFAIRFIQGINSLP-SERHFWSKNPILGNIKVQNVMSRNRYLKINTYLHFNDSSTALP----
REDVNHDKLHHIRPLIDRLAETFAAQYMPSRENAIDEGLVKFKGR-LGFKQYMPMKPIKRGIKAWMRADSITHFVSRFQVYTGRPRG--------------GQ-
EHGLGERVVTELSS-DLEDGYY--HLYFDNFFSTFRLMKSLLEK--GIYATATTRPNRKGFPAPLKN------AKLSRGESLVMQKSGITACSWQDK--
KKVNFFTTN--------CQPNGQGTVQRRS-RDGTLTDVNAPPCVSAYNKFMGGVDYADQKRGDYKIPI-KSRRWYRYLAIFFIE-TAAVNAFILR---QLSPN---
--------HARTTQLNF----RLELINNLLGN--YCSR----------KRSAGVMEVADGKTHFPVKGPIN----RCMQCAKTG------QRH-------
RSSWRCALCDVTLCVG-CFERFHSGQH------------------------------
>AmiPB3
---------------------------------------------------------MAASLVQPMEISREEYDNIFGSSDEEMSNEDSDIDVSELEDESD------
--------ENDESGSESASGSESESDEAVQWTDRLQNIHLEDFTSP-----------VGITFEIGNEARELDVLKKLFNDEILNVIVRETNRHAR------
QKLAGDA-LDKWQDVTLEEIKAFLGVSVVMGVNILP-SISDYWSSNQFLGNEGIQKVMTKNRYENISRFFHFNDSSVEPR----
RGEDGYDRLYKVRPILSHFNAKIQEIYKPGKNISVDEGMIGFKGR-LSFRQYMPAKPTKYGIKVWMAADASNGFVINHEVYLGKQRG--------------
RVLANGLGYSVVMELMN-PFLNKNH--HVYFDNFFSSPKLLEDLQNE--GTYACSTVRAGRVGLPPSSRR------KLKREGEMICEQKGNLVYTKWHDK--
RDVNILSTN--------FDPLEPKTVKERWKKNGDVVLVEKPACVDLYNTSMGGVDRTDQLRSYYSACR-PSKKWYKYLFWFIFD-VSLVNSFII--FKENVDR---
--------RGRRTLVNF----RLALATQLIAG--FSSRSENRKRTMKAASIEATTTPENAPGHFIIRREGNARKRHCVQCKKDGIKTPSNRAK-------
ETIYECAQCGIALCKDPCFLRFHSL--------------------------------
>AmiPB4
-----------------------------------MIHADQGSDFDIESSSDE-----NEEYHNQQSFSESSESQEMSENDNQSINNED-QANARGRGHG-------
-------QGAARGNRTSRHGQQEDALLQIQWTSNDRQPIIPQFTGR-----------
QGLQVQRPNNAGAGENLSLFLTDEFFDLLVEQTNLYVAQYKASNPNLPPHSRASSWVETTRNEMKKFLALSFLIVVVRKL-
EVSGCWSTNPLLKGSIFHNVMPRNCFQSILQFLHFADNSYYN-----ATDPNCDRLYKVRPIVQDLVSKFKSVYIPEEHISIDEELLLWKGK-
LLFKQYIALERARFGIKMFSLCET-TGYLRNSYVYLGKEP-------DAIATAMEMVSRLGKSGAVIPRLME-GLLGFG----------TLAGNLFFPLYEN--
ETVACGTARKNRLMLPASMMTP---RLAK---VEHVFRRKDDMLAVRLNDK--KEIYFLTTMH------------KANVADTGKRDGCGNNVRKLQLVHDYNK-
```

MGGVEHNDELLRTYYCVR-KSMKWIKKVAFHFIE-KSLLNPHNLYKK--SGGRK--------------PLLKF----KLECISALLAA----SSTDTMAPDGSN---
--QFSGQHFPELIPPTPKKQTPQKKCQVCSKEGRRKQSR-----------YQCGQCNTQPGLCAAPCFKIYHTQ--------------------------------
>AmiPB5
---MTAFSDEIASNSRQNTVQIQFVDEEDLPLASFFERSESEDEVDTDFEESEE---------
--------EEEENLSVSEPGDDADVVEISEWRREINLREELAFDQP-----------TGRRIDVRGDMKSVDFFQLFFTDGVWNLIVEQTSLYAE-------
QKREPGERSVWYPLTVSEFKAWIALTLDMGILKKP-SLRLYWSTDSILKTSFFPSVMARDRYFQILRYLHFADNRDEVKD---
NDSPGYDKLFKITKLLDVLLPRFTDVYSPERNLAVDETLIKFKGR-VHFRQFIPIKPGRFGIKCFTLAESSSGYGLVSKVYTGKENG---------------
VVQTDLGRRAVMTLME-SFLDKGY--VLYMDNYYTSVPLFEDLERR--GTLACGTVRSNRKGLPKDITDPKNAEVKALERGDSLYRQKGTVTCVGWKDS--
KMVYMLATTPV-----DPTLNSEVERSVKVDNKWQKKVVNQPSVIYSYNQNMGGVDLSDQRVTTYSRLM-KGSVWCLKIFFYLLE-LSMSNAQIIMAKSFGNDAP--
------------AMLEF----RRNVIKELVNG-------KTFRLTEGNPVPAAPIPQFRFNREYFHYPIANNSRLACKVHIQRVDTN----------------
YSCAVCGVSMCPHPCFYRYHTMVDYLFNDESRNGPRRLSEGRGRPRQLRRSTEH
>AmiPB6
--MSSLVFVLPPLSSFRSSIEEEEEEIN-----LRTVQIEE------
--------TDDSEEELAKGKPSNVTVEEFSWKDNRTEIDIPGFSQA-----------VGPTTILPVGILALDFFLLFMSNRILQNILRETNRYAS------
QTLQRIKILTTWKQLSMEELKAFFGLLVAMSIHELP-CLQDYWSSDWVLSVPAFSRVMPLNRFLDIWNNVHLSDNTKMPK----
PGNSNFDKLFKVKQFIDDLKTNFQINYAPHREQAVDEAMIKYKGR-TYLKQYMPMKPIKRGIKMWCRADSTNGYLCDFDIYTG-KTQ--------------
QGVQHGLDYSVVTKLCE-TIKGHWY--AIFCDNFFTSYKLIEDLYRS--KILCCGTLRSGHKEFPPCLFDKD--RIKSMKRGDVFWQMKGPVFAITWMDK--
KAVHAAGTYT-------QAPQENLPEVNRKQKDGTLQRITYPQLISSYNTYMAGVDKNDQMKSYYTIPV-SGKKWWSRILFDLVD-RSIFNSFVLE---QESPN---
--------HAKRTLKSF----RIDLAKQLIGD--FSSQRKRGRP--SDEPLCLRNVERHFPDYLPTNESGKRKERRCKVCFNSG------VRK-------
MTSYFFPDCDVGLCATPCFRTYHQ---------------------------------
>NvePB1
---MAESLAIDASEFRAIFGVNPDENDDVNNSENEFQGFELSS------
--------EEESSSSEEESEDESDEDSVKNWSEQLTHFDLEDFTGQ-----------QRLKKTVSEDAKAEDYFSLMFSDELIGLISDETNRYAQFII--
GQKADREARLAKWKDVTPDELRMFLGVALVMGINSLP-QQALYWSQNPLFGNLGIQDVIPKTRFEEIAQFLHFSDPNNEPQ----
RGDADYDRLFKVRPVLSDVLKSIQQAYKPSKDISVDEGMIAFKGR-LGFRQYMPAKPTKYGIKVWMAADANNGYVCNFKVYLG-QEN--------------
RQRVHGLGYDVVMDMAK-PFLNSYR--HIFCDNFFTTVRLFDHLLDQ--KTYACGTVRVNRRELPPCAKE------KLK-PGEMLQRQRGQLLFTKWHDK--
RDVSLLSTN--------VSPLEPGRTVRRKYRSREEIDIEKPKVTDVYTAHMGGVDRADQLRSFYSSGW-QSRKWYKYMFWFLFN-LAVTNAFVLEGFKRQLQG---
--------KAKRTVIVF----KTELAQSLIAK--FNSR--------KRPRVPAPPTPQNIPASAHVATHVEGRKRACVLCSKKGKTTPKGYKI-------
ETYYECAVCKVGLCRKGCHQAFHEMDHGTA---------------------------
>NvePB2

--MTRAVDFFQLFFTAHLVQQISANTNAYAYANI--
EQKQSYANEMGAWNDTNPEEINRLIALILYCGLVNVS-SFHRYWNTKTLYHGLWARAIMARDRFKALMAMLHVVNPIEEDD---------
HDKLRKVRSFLDHFKERCKALYQPFQNVAIDEKMVKSKHR-SGMRQYIKNKPTKWGLKLWVLADSTNCYTYDFDVYAGRQTAN-------------
APSTNGLAYDVVMKLLS-PLLNQGY--HLYCDNFYTSVTLIKDLFLV--KTPATGTATENRRGFPEEMKNGR-KWAGKQERGSMRWHRDGVCLAQQWKDN--
RPVTILSSIEC-----ANDYVIVSRKVKS-REQWTNIEVRQPMAINNYNSYMNGVDRSDQLMTKNNALS-KCRRWWKVLFFHMID-IAVVNSYILFQLHRAQNP---
-DNTALYRPKKYSVAEF----REELVRQLVGL--EEFGPPP------AHKPPTRAPNQFETVHMPMMSDVKRNCKVCYATTGKELKVRTYCEA-------P------
QCQAYLHLTSTQNCFKIWHSKEFHE-------------------------

```
>NvePB3
MASVRVRRNRQRNLTVDEVLSSVFDENTTDLDSSDSEDGEDELPTLFPAIPADLEDDSVESVSSYEDSSSSSSDSEFDRRQSVTNQRQEKARNLRECSQNARRTIRG
VRRGRGARRGRAISVQQRREIQDGGRQQYIWSKEVNRRILKPFTNAVG----------LANRGQRREKSALEYFELFFDQEVWDCLVTMTNLNAE------
RKGAKGTSGGQWKPITQDEMKAFFGLNIAMGIVKLPEAKMYWQKKIWLLTVPSFSQVMSRNRFFQILQYIHVSDDSNIVP----
SGQPGYDKLHKIKPLLNLLFPKFENAYNLHKNISIDECMIPWRGR-LSFRQFIANKPVRFGIKVWVLADSESKYVYRQQLYIGKNPG--------------
ERAEVGLAARVVKELCS-GLEGMGH--HLYTDNYYTSVELYQFLFDN--QIYACGTIKGNRKNFAKEVLFEN---TRGLARGSSKWLMCGPLLAVGWLDN--
KAVYFLSTIHPPEFPVRAPVHARNVKRRGAGEGGQSGDIPCPPLLKDYNAYMGGVDQSDQMLRYYTCIR-KTVKWYRRVLFHEVE-VAIHNAFVLECEDREGTPR--
---------PIRTSLEF----REELAENLIGN--
MRVAHNAAQRPPRNEELRLTDVGLHLPEMRQDKGNCAVCSKKIRKTHSDRYKDVPLARRPKVPYVPRPSIYCNVCNVFLCLQKDQNCWKDFHTKVEYWR--------
----------------
>TruPGBD2
---------------MQHVKSLIISFIYREDTSYEDSSEEEWNSSATKRGAVGKHRCSDSVSPLSMSLRSPSKRKEERCMCCPGPPRKRISNASTLPKVSS------
--------SLQAVGVFSSGEKQAEKELARNEPDADRWHDMDEEDFEPPQPRFRPERVPGPQLNRTANYTPLELFQLFFSTTVIDNLVKHTNANG--------
KKKRQAQQKHWHTVTREDMYSFLSLVLYMGMVPLK-
AMTDFWKGSKLYRLPFPSSVMPSNRFLAISRSLHINDPAVETANDLKKGTPGYDKLCRIKPLYEQILAACYTFFHPYQHISVDERMVATKAR-
IGLKRYVRNKPTKWGIKLFVLADSSCGYTLNFFVDAGKDP---------------EPTGKGRSYDIVMRILNVPFLGKGY--KLYMDNFYTSPTLFRDLLSR--
KIWACGTLRPNMAGYPK--KKGN-DMTDKTPRGTIRWMRQGELLFVKWLDA--RQVNMCSTMHK-----
AYSNDTAMRKVKNAKGEWTVKRVPIPGCIKDYNQHMGGVDLSDALISYYNVLH-KTLKWYKTLFYHFVD-
IATVNAFILHKEMCKLQDRPELKQKAFREQLILSLAGIGSTPRRSAPQNHPGL--AVAAPAAP---
SSSQVPDTTPPTAPASSHLPAYFVEHMNDVTPRFRATLGRRTCVLCKR-------KSPVYCSTCKKTLCFTSLRNCYRDWHRSNSICT-------------------
-----
>HsaPGBD4
------------------------------------MSNPRKRSIPMRDSNTGLEQLLAEDSFDESDFSEIDDSDNFSDSALEADKIRPLS----------------
----------HLESDGKSSTSSDSGRSMKWSARAMIPRQRYDFTGT-----------
PGRKVDVSDITDPLQYFELFFTEELVSKITRETNAQAALLASKPPGPKGFSRMDKWKDTDNDELKVFFAVMLLQGIVQKP-
ELEMFWSTRPLLDTPYLRQIMTGERFLLLFRCLHFVNNSSISA----GQSKAQISLQKIKPVFDFLVNKFSTVYTPNRNIAVDESLMLFKGP-
LAMKQYLPTKRVRFGLKLYVLCESQSGYVWNALVHTGPG------------MNLKDSADGLKSSRIVLTLVN-DLLGQGY--CVFLDNFNISPMLFRELHQN--
RTDAVGTARLNRKQIPNDLKKR---IAK----GTTVARFCGELMALKWCDG--KEVTMLSTFH------------NDTVIEVNNRNGKKTKR--
PRVIVDYNENMGAVDSADQMLTSYPSERKRHKVWYKKFFHHLLH-ITVLNSYILFKKDNPEHTM--------------SHINF----
RLALIERMLEKHHKPGQQHLRGRPCSDDVTPLRLSGRHFPKSIPATSGKQNPTGRCKICCSQYDKDGKKIRK-------
ETRYFCAECDVPLCVVPCFEIYHTKKNY-----------------------------
>AgaPB8
----------------------------------------------MFEANMPKLIEAQSGDEEDIRIPKKNTRNRIVSSDSESDNYDGSDCDCDESSLPDVFR---
------EMELEEERNHEGLPQQKSTIQSTAWSEFTGRQKSFAFVEE------------NQLSLPPLDMTPYNVFQLFVDQEIIEMIVTQTNKYALQKLD-
NATLSQGARMSKWKPTNSAEMKKFLSLLLWMGLVKVN-PVVNYWSKNTLYNFKLPSTVMSRNRFEILLSNLHFTDNTSISP---------
KNRQGKIINLMDKLQEKYQKVYTPGENIVIDETLIPWRGR-LIFRQYIPSKAHKYGIKMFKLCSS-EGFTWASKIYSGKSSE--------------
GIRETGLAHVCIKLAEK--LFEKGR--TLYVDNLYTSYELALTCLDR--KTHLVGTLRHNKKSMPRDVLDCK------LKKGEMIX---VGIVVLKWKDS--
RDVRMLSTKHAP----------YMVQTTKKCYSNQPATKLKPLTVVDYNKGKCGIDYSDQMVSYATTMR-EGVKWYRKLGIELLLGTSVVNALVVFKTATKRNIG--
```

-------------IREF----REMIASELLQLP--------------
VKNTTKXSATRKSCFSHPSRFQWEKMRRACKRCYNNDRQSMGRSKVRETVKRSFTNCPSCPDQPQLCDECFKIVHQK------------------------------
---
>TniPB
---------------------------------MGSSLDDEHILSALLQSDDELVGEDSDSEISDHVSEDDVQSDTEEAFIDEVHEVQPTSSGSEILDEQNVIEQP-
------GSSLASNRILTLPQRTIRGKNKHCWSTSKSTRRSRVSALNIVRS------QRGPTRMCRNIYDPLLCFKLFFTDEIISEIVKWTNAEIS------
LKRRESMTGATFRDTNEDEIYAFFGILVMTAVRKDN--HMSTDDLFDRSLSMVYVSVMSRDRFDFLIRCLRMDDKSIRPT------
LRENDVFTPVRKIWDLFIHQCIQNYTPGAHLTIDEQLLGFRGR-CPFRMYIPNKPSKYGIKILMMCDSGTKYMINGMPYLGRGTQ---------------
TNGVPLGEYYVKELSK-PVHGSCR--NITCDNWFTSIPLAKNLLQEPYKLTIVGTVRSNKREIPEVLKNSR-----
SRPVGTSMFCFDGPLTLVSYKPKPAKMVYLLSSCD--------------------EDASINESTGKPQMVMYYNQTKGGVDTLDQMCSVMTCSR-
KTNRWPMALLYGMIN-IACINSFIIYSHNVSSKGE-----------KVQSRKKFMRNLYMSLTSSFMRKR---
LEAPTLKRYLRDNISNILPNEVPGTSDDSTEEPVMKKRTYCTYCPSKIRRKAN--------------ASCKKCKKVICREHNIDMCQSCF-----------------
----
>Pigibaku1
----------------------------------MAKRNSAQRALELILEDREVFDDDVEGEDSEEEVSEFEDHISENSESDSDSEEDDEIEHQPVPKQRRATGPG-
---RQQPSPGPEQASQHGGEIWMSKNSEIEWSSRPRKGPPHKAANVIR--------MQPGPDMAVTHTRDIKSFELFIPDSIQEIILDCTNLEGR-----------
RVFGERWKELDQTQLHAYFGVLILAGVFRSK-GESAESLWDAETGREIFRATMSLENFHIISRIIRFDN-DDRPAR------
WQRDKLGVIRTVWDKWVRRLPLLYNPGPNVTIDEQLMPFRGR-CPFLQYLPSKPAKNGIKIWAACDATSSYAWNLQVYTGKPDGG-------------
APEKNPRNESCPRHVSG----TQWT--QHHMRHFFTSHKLGQELLKR--KLTIVGTIRKNRSELPPQLLTSK-----
NRPVKSSQFAYTADTSLVSYVPKKGKNVVLMSTLHR-------------------DGRMCDQEHHKPEIIMDYNATKGGVDNMDKLVTAYSCKR-
RTLRWPLVIFFDMLD-ISAYNAFVIWMALNPEWKR--------------
VKLQKRRLFLEDLGKALVRPQIERRKHIPRTPASAAMVRRIQKENAGALSTQPTEPQSAEPEVNV-XVVNSSNKKKRCEVCG-
PKMDRKTQYTCIKCKKYICNTHTVKLWPSCVV------------------------------
>BmoPB1

ADIIAYIVRETNRYGAQLIRSTIPSKPNSRLSRWKDVTSDEIYGFXVLMLQSLVIDYV-EREYWYAVIEELQIGNFKEIMTYNRFIVIKRCLHFIDNATLPV----
PPT----KLDKIIPIIEHLNKKFKSLYVLEQNIAIDESLLLWKGR-
LSFAQKIATKRPRVGIKSYELCESRTGYLWQMEVYTGKGHAHVVQDGEPEERGQESDEPESATAQIVLNLTR-PLFDKGH--TLIMDNFYNAPLLSRILKVQH-
KTDSMGTLRLNREFVPEALKKK---TKKNMKEGEVAFSTTKDLSVVVWMDK--NIVAMISSFH------------PIEVGGIEK-YGYYRYK--
PQVVLDYNFSMGGIDHKDQMLSAYPIERSRNIIWYKKLFRRLLN-VSMHNALVMFN-HNRTHAL--------------RHREF----
RLQLAKQLLQSSRSAAPSRS-LAPAARPEPAACIRDMHLP-------GKNTKKQRCKLCYSAKVQR--------------STVWRCTLQCHLCIV------------
----
>XtrKob_DB_620

MAKRVYSTGKTDRVCMMNSSSEEFSDYDSEYEPFGSETDFSTDESEIGDSSSSVSAEVSCSGSDIADRAEASFSESVNLPEVPTEENTAEDEVTIVDAVVGSEPMWS
PPNNYSPEIPLFTAV-----------PGVKVNTNN-FELVDFFHVFVTEDILQNMVFYTNLYAEQFLARCP-LPEYSQGHAWHPTNTHEIKRFLALTIAMDLHEGH-
TLASYWDTTSIISIPLFSAIMPINRYQILLRFLHFNDNATAAP----PNEPSHDRLHKLRPLIESLCKRFAEVYTPTQNICVNESPVFFKER-

LMFRQNNPKKRSCYGMKFYKLCESKTGYTGNFMIYEGT---------DSFLDPPGCPLDFTFSDKIVWELLT-PLLGRGY--HLYVDDFYTSIPLFRALHSL--
DTPACGIVSHNRKGLPRALINK---ILRH---GETYALRSNDLLAIKYCDT--KNEYILTTIH------------DESVIVE-
HKTTNGPPKSKPLCSKEYSRHIDGVNKTDQIQ-YYNAAR-KTKAWYKKAAFYMIQ-MALNNSYVVYKATVPGPKL--------------SFYNY----
QVQLLPSLLFG----DVEE--APDMSDNDNVVRMVGKHFIDNIPPTLNKKYAQRACKVCRKKGIRRDVR-----------
YYCPKCPSKPALCFQPCFEIYHTVVHYEKA--------------------------
>XtrUri1_PCR
-------------------------------MAKRFYSTEEAARYCMD-SSSEEFSDSGSEYVP----SDHSTEESEIEDSD-TVSAEFSSGGTLTAEEAEASGSA-
---QDVPMDVEEGEGST---DAAVG-EPAWGPPCNYVPEIPPFTAV-----------PGLSVDTTN-FEIIDFFNLYITEAILQDMVRYTNLYAEQYLASNP-
LPGFSRAQAWYPTNINEIKRFLALTLAMGLVERN-SLASYWDTNTVLSIPLFSAVMARNRYQILLRFLHFNDNTTAVA----
PNEPGYDRLYKLRPLIDSLSQRFAEVYTPSQNVCVDESLLLFKGR-LKFRQYIPSKRSRYGIKFYKLCESSTGYTSYFMLYEGK---------
DTNLDPPGCPTDLTTSGKIVWELIT-PLLGRGY--HLYVDNFYTGIPLFRALNSL--DTPACGTVHRNRKGLPRELLDK---KLKR---
GEVHALRSNELLAIKYSDT--KVVLMLTTIH------------DETMIVQ-HRSGGRPSKTKPLCSKEYSKHMGGVDKSDQIQHYYNATR-KTRAWYKKAAIYMIQ-
MALYNSYVVYKAAVPGPRL--------------SYYNY----LLQLLPALLFG----DVEE--
VPDMPGNDNVARMVGKHFIAQIPPTPNKRYAQRKCKVCRSKGVRRDVR-----------YYCPKCPSKPALCFHPCFEVYHTVVH----------------------
----
>XtrUri2_DB_469b
-------------------------------MAKRFYSAEEAAAHCMA-SSSEEFSGSDSEYVPPASESDSSTEESWCSSSTVSALEEPMEVDEDVDD---------
-------LEDQEAGDRA---DAAAGGEPAWGPPCNFPPEIPPFTTV-----------PGVKVDTSN-FEPINFFQLFMTEAILQDMVLYTNVYAEQYLTQNP-
LPRYARAHAWHPTDIAEMKRFVGLTLAMGLIKAN-SLESYWDTTTVLSIPVFSATMSRNRYQLLLRFLHFNNNATAVP----
PDQPGHDRLHKLRPLIDSLSERFAAVYTPCQNICIDESLLLFKGR-LQFRQYIPSKRARYGIKFYKLCESSSGYTSYFLIYEGK---------
DSKLDPPGCPPDLTVSGKIVWELIS-PLLGQGF--HLYVDNFYSSIPLFTALYCL--DTPACGTINRNRKGLPRALLDK---KLNR---
GETYALRKNELLAIKFFDK--KNVFMLTSIH------------DESVIRE-QRVG-RPPKNKPLCSKEYSKYMGGVDRTDQLQHYYNATR-KTRAWYKKVGIYLIQ-
MALRNSYIVYKAAVPGPKL--------------SYYKY----QLQILPALLFG----
GVEEQTVPEMPPSDNVARLIGKHFIDTLPPTPGKQRPQKGCKVCRKRGIRRDTR-----------YYCPKCPRNPGLCFKPCFEIYHTQLHY---------------
----
>HsaPGBD1
------------------------------ECAPQIPCSTPIATERTVAHLNTLKDRHPGDLWARMHISSLEYAAGDITRKGRKKDKARVSELLQGLSFSGDSDVE-
-------KDNEPEIQPAQKKLKVSCFPEKSWTKRDIKPNFPSWSALDS----------GLLNLKSEKLNPVELFELFFDDETFNLIVNETNNYAS------------
-QKNVSLEVTVQEMRCVFGVLLLSGFMRHP-RREMYWEVSDTDQN-LVRDAIRRDRFELIFSNLHFADNGHLDQ---------
KDKFTKLRPLIKQMNKNFLLYAPLEEYYCFDKSMCECFDS----DQFLNGKPIRIGYKIWCGTTT-QGYLVWFEPYQEESTMKVD-----------
EDPDLGLGGNLVMNFADVLLERGQYPYHLCFDSFFTSVKLLSALKKK--GVRATGTIRENRTEKCPLMNVEH-MKKMKRGYFDFRIEENNEIILCRWYGD--
GIISLCSNAVG--------IEPVNEVSCCDADNEEIPQISQPSIVKVYDECKEGVAKMDQIISKYRVRI-RSKKWYSILVSYMID-VAMNNAWQLHRACNPGASL--
--DPLDFRRFVAHFYLEHNAHLSD---------------------------------

>CinPB2
---MMQLQLILSGDVSDVEDIGSSSEDETDNTVQESFQDTVMN------
------------ISVPDKVESEHKKKHVYRWRKRNIPVTDSNFQLQ--------------KDNIDSIHSPITYFMQFFPESLIRTIVEQTNLYST------------
QKIGVSINTNYSEICMFIAIQIKMSILHLP-SYLMYWS--KEMRFPPIADSISLKRYQKLRRFLHFVDNSTFNS-------DNPNKLFKIQPVLDAVRNECIKIQ-

```
PEQSHSVDEQIIPAKTKYSKFRQYNPKKPVKWGFKNMVRADS-SGFMYDFYIYGGKDSKQ--------LALSDDATHLQKSAQTVVKLCQHLPVKQNH--
QLFFDNWFTTLDLLIYLKEI--GINACGTIRGN-----EDLKKNG------RGAVDFRSDFNSGILVTKWYDN--NAVHIGSNFVG--------
IEPMSTIDRWSSEIKEKVPIQCPQIITMYNKGMGGVDLADMLISLYRTEV-KTRRWYIKIFWHCID-IAKVNAWLLYRRHCNEMSIS--------KKSQRTLLNF--
--VVDISDNLLYRN-SCSTPTRGRPSNKRKLENDTLPGKKPFTPTPNEILRFDQISHWPEPRSQRGRCRLCKSG-------
YSQIYCSKCHVCLCLKSSNNCFKEFHCKN----------------------------
```

B. “aligned” format

```
AmiPB1              ----------------------------------------MFSLHYTGPTDTAAFYGAAA 20
AmiPB2              -----------------------------------------------------------M 1
AmiPB3              ---------------------------------------------------------MAA 3
AmiPB4              -----------------------------------MIHADQGSDFDIESSSDE-----NE 20
AmiPB5              -----------------------------------------------MTAFSDEIASNSR 13
AmiPB6              ------------------------------------------------------------
NvePB1              ------------------------------------------------------------
NvePB2              ------------------------------------------------------------
NvePB3              MASVRVRRNRQRNLTVDEVLSSVFDENTTDLDSSDSEDGEDELPTLFPAIPADLEDDSVE 60
TruPGBD2            ---------------MQHVKSLIISFIYREDTSYEDSSEEEWNSSATKRGAVGKHRCSDS 45
HsaPGBD4            ------------------------------------MSNPRKRSIPMRDSNTGLEQLLAE 24
AgaPB8              ----------------------------------------------MFEANMPKLIEAQS 14
TniPB               ---------------------------------MGSSLDDEHILSALLQSDDELVGEDSD 27
Pigibaku1           ----------------------------------MAKRNSAQRALELILEDREVFDDDVE 26
BmoPB1              ------------------------------------------------------------
XtrKob_DB_620       -------------------------------MAKRVYSTGKTDRVCMMNSSSEEFSDYDS 29
XtrUri1_PCR         -------------------------------MAKRFYSTEEAARYCMD-SSSEEFSDSGS 28
XtrUri2_DB_469b     -------------------------------MAKRFYSAEEAAAHCMA-SSSEEFSGSDS 28
HsaPGBD1            ------------------------------ECAPQIPCSTPIATERTVAHLNTLKDRHPG 30
CinPB2              ------------------------------------------------------------


AmiPB1              ARPLPRVPAGPRAPPVVPVLARAGRALGRARGGRAQARGGR--------------RGRAR 66
AmiPB2              ASVSDIFDSDSEEEEFFGFSTNDIARADISGE----SDISA--------------DESFT 43
AmiPB3              SLVQPMEISREEYDNIFGSSDEEMSNEDSDIDVSELEDESD--------------ENDES 49
```

```
AmiPB4           EYHNQQSFSESSESQEMSENDNQSINNED-QANARGRGHG--------------QGAARG 65
AmiPB5           QNTVQIQFVDEEDLPLASFFERSESEDEVDTDFEESEE-----------------EEEEN 56
AmiPB6           --MSSLVFVLPPLSSFRSSIEEEEEEIN-----LRTVQIEE--------------TDDSE 39
NvePB1           -MAESLAIDASEFRAIFGVNPDENDDVNNSENEFQGFELSS--------------EEESS 45
NvePB2           ------------------------------------------------------------
NvePB3           SVSSYEDSSSSSSDSEFDRRQSVTNQRQEKARNLRECSQNARRTIRGVRRGRGARRGRAI 120
TruPGBD2         VSPLSMSLRSPSKRKEERCMCCPGPPRKRISNASTLPKVSS--------------SLQAV 91
HsaPGBD4         DSFDESDFSEIDDSDNFSDSALEADKIRPLS--------------------------HLE 58
AgaPB8           GDEEDIRIPKKNTRNRIVSSDSESDNYDGSDCDCDESSLPDVFR---------EMELEEE 65
TniPB            SEISDHVSEDDVQSDTEEAFIDEVHEVQPTSSGSEILDEQNVIEQP-------GSSLASN 80
Pigibaku1        GEDSEEEVSEFEDHISENSESDSDSEEDDEIEHQPVPKQRRATGPG----RQQPSPGPEQ 82
BmoPB1           ------------------------------------------------------------
XtrKob_DB_620    EYEPFGSETDFSTDESEIGDSSSSVSAEVSCSGSDIADRAEASFSESVNLPEVPTEENTA 89
XtrUri1_PCR      EYVP----SDHSTEESEIEDSD-TVSAEFSSGGTLTAEEAEASGSA----QDVPMDVEEG 79
XtrUri2_DB_469b  EYVPPASESDSSTEESWCSSSTVSALEEPMEVDEDVDD----------------LEDQEA 72
HsaPGBD1         DLWARMHISSLEYAAGDITRKGRKKDKARVSELLQGLSFSGDSDVE--------KDNEPE 82
CinPB2           -MMQLQLILSGDVSDVEDIGSSSEDETDNTVQESFQDTVMN------------------I 41


AmiPB1           --ARGGGQVRGAAVTLHETYDVRDVGNPPLPFNPTRP---IGVHFGQRLLRNAMTTAVEF 121
AmiPB2           EDEESDSNSVESGGEEDWSDDLDFVIVEDFNKP-----------TGPTTVLPATATAGDF 92
AmiPB3           GSESASGSESESDEAVQWTDRLQNIHLEDFTSP-----------VGITFEIGNEARELDV 98
AmiPB4           NRTSRHGQQEDALLQIQWTSNDRQPIIPQFTGR-----------QGLQVQRPNNAGAGEN 114
AmiPB5           LSVSEPGDDADVVEISEWRREINLREELAFDQP-----------TGRRIDVRGDMKSVDF 105
AmiPB6           EELAKGKPSNVTVEEFSWKDNRTEIDIPGFSQA-----------VGPTTILPVGILALDF 88
NvePB1           SSEEESEDESDEDSVKNWSEQLTHFDLEDFTGQ-----------QRLKKTVSEDAKAEDY 94
NvePB2           -----------------------------------------------------MTRAVDF 7
NvePB3           SVQQRREIQDGGRQQYIWSKEVNRRILKPFTNAVG----------LANRGQRREKSALEY 170
TruPGBD2         GVFSSGEKQAEKELARNEPDADRWHDMDEEDFEPPQPRFRPERVPGPQLNRTANYTPLEL 151
HsaPGBD4         SDGKSSTSSDSGRSMKWSARAMIPRQRYDFTGT-----------PGRKVDVSDITDPLQY 107
AgaPB8           RNHEGLPQQKSTIQSTAWSEFTGRQKSFAFVEE------------NQLSLPPLDMTPYNV 113
TniPB            RILTLPQRTIRGKNKHCWSTSKSTRRSRVSALNIVRS------QRGPTRMCRNIYDPLLC 134
Pigibaku1        ASQHGGEIWMSKNSEIEWSSRPRKGPPHKAANVIR--------MQPGPDMAVTHTRDIKS 134
BmoPB1           ------------------------------------------------------------
XtrKob_DB_620    EDEVTIVDAVVGSEPMWSPPNNYSPEIPLFTAV-----------PGVKVNTNN-FELVDF 137
XtrUri1_PCR      EGST---DAAVG-EPAWGPPCNYVPEIPPFTAV-----------PGLSVDTTN-FEIIDF 123
XtrUri2_DB_469b  GDRA---DAAAGGEPAWGPPCNFPPEIPPFTTV-----------PGVKVDTSN-FEPINF 117
HsaPGBD1         IQPAQKKLKVSCFPEKSWTKRDIKPNFPSWSALDS----------GLLNLKSEKLNPVEL 132
```

```
CinPB2               SVPDKVESEHKKKHVYRWRKRNIPVTDSNFQLQ--------------KDNIDSIHSPITY 87


AmiPB1               FNLFFTVELVNNIVTHTNSYAYEHI--VTHQSYAKSDGSWQEATTDEIRRLIAILIYFGL 179
AmiPB2               LNLVFPEDLIQLIVDETNRNAR-----QKQQQSGTVDKDWMPVNNSDIKAFFAIRFIQGI 147
AmiPB3               LKKLFNDEILNVIVRETNRHAR------QKLAGDA-LDKWQDVTLEEIKAFLGVSVVMGV 151
AmiPB4               LSLFLTDEFFDLLVEQTNLYVAQYKASNPNLPPHSRASSWVETTRNEMKKFLALSFLIVV 174
AmiPB5               FQLFFTDGVWNLIVEQTSLYAE-------QKREPGERSVWYPLTVSEFKAWIALTLDMGI 158
AmiPB6               FLLFMSNRILQNILRETNRYAS------QTLQRIKILTTWKQLSMEELKAFFGLLVAMSI 142
NvePB1               FSLMFSDELIGLISDETNRYAQFII--GQKADREARLAKWKDVTPDELRMFLGVALVMGI 152
NvePB2               FQLFFTAHLVQQISANTNAYAYANI--EQKQSYANEMGAWNDTNPEEINRLIALILYCGL 65
NvePB3               FELFFDQEVWDCLVTMTNLNAE------RKGAKGTSGGQWKPITQDEMKAFFGLNIAMGI 224
TruPGBD2             FQLFFSTTVIDNLVKHTNANG--------KKKRQAQQKHWHTVTREDMYSFLSLVLYMGM 203
HsaPGBD4             FELFFTEELVSKITRETNAQAALLASKPPGPKGFSRMDKWKDTDNDELKVFFAVMLLQGI 167
AgaPB8               FQLFVDQEIIEMIVTQTNKYALQKLD-NATLSQGARMSKWKPTNSAEMKKFLSLLLWMGL 172
TniPB                FKLFFTDEIISEIVKWTNAEIS------LKRRESMTGATFRDTNEDEIYAFFGILVMTAV 188
Pigibaku1            FELFIPDSIQEIILDCTNLEGR-----------RVFGERWKELDQTQLHAYFGVLILAGV 183
BmoPB1               ------ADIIAYIVRETNRYGAQLIRSTIPSKPNSRLSRWKDVTSDEIYGFXVLMLQSLV 54
XtrKob_DB_620        FHVFVTEDILQNMVFYTNLYAEQFLARCP-LPEYSQGHAWHPTNTHEIKRFLALTIAMDL 196
XtrUri1_PCR          FNLYITEAILQDMVRYTNLYAEQYLASNP-LPGFSRAQAWYPTNINEIKRFLALTLAMGL 182
XtrUri2_DB_469b      FQLFMTEAILQDMVLYTNVYAEQYLTQNP-LPRYARAHAWHPTDIAEMKRFVGLTLAMGL 176
HsaPGBD1             FELFFDDETFNLIVNETNNYAS-------------QKNVSLEVTVQEMRCVFGVLLLSGF 179
CinPB2               FMQFFPESLIRTIVEQTNLYST------------QKIGVSINTNYSEICMFIAIQIKMSI 135
                                 :   *.                            ::     : .   .

AmiPB1               VRVSDAADNYWSIQSPYLGLWGKCFLSRIRFKALMAMLHVVDPANEPD---------GDK 230
AmiPB2               NSLP-SERHFWSKNPILGNIKVQNVMSRNRYLKINTYLHFNDSSTALP----REDVNHDK 202
AmiPB3               NILP-SISDYWSSNQFLGNEGIQKVMTKNRYENISRFFHFNDSSVEPR----RGEDGYDR 206
AmiPB4               VRKL-EVSGCWSTNPLLKGSIFHNVMPRNCFQSILQFLHFADNSYYN-----ATDPNCDR 228
AmiPB5               LKKP-SLRLYWSTDSILKTSFFPSVMARDRYFQILRYLHFADNRDEVKD---NDSPGYDK 214
AmiPB6               HELP-CLQDYWSSDWVLSVPAFSRVMPLNRFLDIWNNVHLSDNTKMPK----PGNSNFDK 197
NvePB1               NSLP-QQALYWSQNPLFGNLGIQDVIPKTRFEEIAQFLHFSDPNNEPQ----RGDADYDR 207
NvePB2               VNVS-SFHRYWNTKTLYHGLWARAIMARDRFKALMAMLHVVNPIEEDD---------HDK 115
NvePB3               VKLPEAKMYWQKKIWLLTVPSFSQVMSRNRFFQILQYIHVSDDSNIVP----SGQPGYDK 280
TruPGBD2             VPLK-AMTDFWKGSKLYRLPFPSSVMPSNRFLAISRSLHINDPAVETANDLKKGTPGYDK 262
HsaPGBD4             VQKP-ELEMFWSTRPLLDTPYLRQIMTGERFLLLFRCLHFVNNSSISA----GQSKAQIS 222
AgaPB8               VKVN-PVVNYWSKNTLYNFKLPSTVMSRNRFEILLSNLHFTDNTSISP---------KNR 222
TniPB                RKDN--HMSTDDLFDRSLSMVYVSVMSRDRFDFLIRCLRMDDKSIRPT------LRENDV 240
```

```
Pigibaku1            FRSK-GESAESLWDAETGREIFRATMSLENFHIISRIIRFDN-DDRPAR------WQRDK 235
BmoPB1               IDYV-EREYWYAVIEELQIGNFKEIMTYNRFIVIKRCLHFIDNATLPV----PPT----K 105
XtrKob_DB_620        HEGH-TLASYWDTTSIISIPLFSAIMPINRYQILLRFLHFNDNATAAP----PNEPSHDR 251
XtrUri1_PCR          VERN-SLASYWDTNTVLSIPLFSAVMARNRYQILLRFLHFNDNTTAVA----PNEPGYDR 237
XtrUri2_DB_469b      IKAN-SLESYWDTTTVLSIPVFSATMSRNRYQLLLRFLHFNNNATAVP----PDQPGHDR 231
HsaPGBD1             MRHP-RREMYWEVSDTDQN-LVRDAIRRDRFELIFSNLHFADNGHLDQ---------KDK 228
CinPB2               LHLP-SYLMYWS--KEMRFPPIADSISLKRYQKLRRFLHFVDNSTFNS-------DNPNK 185
                                              :    :  :   .:. :

AmiPB1               LRNINPLLEFFKGRCKDLYQPRQNVAVDERIVKSRHR-SGMRQFNKDKPIRWGIKLWVLA 289
AmiPB2               LHHIRPLIDRLAETFAAQYMPSRENAIDEGLVKFKGR-LGFKQYMPMKPIKRGIKAWMRA 261
AmiPB3               LYKVRPILSHFNAKIQEIYKPGKNISVDEGMIGFKGR-LSFRQYMPAKPTKYGIKVWMAA 265
AmiPB4               LYKVRPIVQDLVSKFKSVYIPEEHISIDEELLLWKGK-LLFKQYIALERARFGIKMFSLC 287
AmiPB5               LFKITKLLDVLLPRFTDVYSPERNLAVDETLIKFKGR-VHFRQFIPIKPGRFGIKCFTLA 273
AmiPB6               LFKVKQFIDDLKTNFQINYAPHREQAVDEAMIKYKGR-TYLKQYMPMKPIKRGIKMWCRA 256
NvePB1               LFKVRPVLSDVLKSIQQAYKPSKDISVDEGMIAFKGR-LGFRQYMPAKPTKYGIKVWMAA 266
NvePB2               LRKVRSFLDHFKERCKALYQPFQNVAIDEKMVKSKHR-SGMRQYIKNKPTKWGLKLWVLA 174
NvePB3               LHKIKPLLNLLFPKFENAYNLHKNISIDECMIPWRGR-LSFRQFIANKPVRFGIKVWVLA 339
TruPGBD2             LCRIKPLYEQILAACYTFFHPYQHISVDERMVATKAR-IGLKRYVRNKPTKWGIKLFVLA 321
HsaPGBD4             LQKIKPVFDFLVNKFSTVYTPNRNIAVDESLMLFKGP-LAMKQYLPTKRVRFGLKLYVLC 281
AgaPB8               QGKIINLMDKLQEKYQKVYTPGENIVIDETLIPWRGR-LIFRQYIPSKAHKYGIKMFKLC 281
TniPB                FTPVRKIWDLFIHQCIQNYTPGAHLTIDEQLLGFRGR-CPFRMYIPNKPSKYGIKILMMC 299
Pigibaku1            LGVIRTVWDKWVRRLPLLYNPGPNVTIDEQLMPFRGR-CPFLQYLPSKPAKNGIKIWAAC 294
BmoPB1               LDKIIPIIEHLNKKFKSLYVLEQNIAIDESLLLWKGR-LSFAQKIATKRPRVGIKSYELC 164
XtrKob_DB_620        LHKLRPLIESLCKRFAEVYTPTQNICVNESPVFFKER-LMFRQNNPKKRSCYGMKFYKLC 310
XtrUri1_PCR          LYKLRPLIDSLSQRFAEVYTPSQNVCVDESLLLFKGR-LKFRQYIPSKRSRYGIKFYKLC 296
XtrUri2_DB_469b      LHKLRPLIDSLSERFAAVYTPCQNICIDESLLLFKGR-LQFRQYIPSKRARYGIKFYKLC 290
HsaPGBD1             FTKLRPLIKQMNKNFLLYAPLEEYYCFDKSMCECFDS----DQFLNGKPIRIGYKIWCGT 284
CinPB2               LFKIQPVLDAVRNECIKIQ-PEQSHSVDEQIIPAKTKYSKFRQYNPKKPVKWGFKNMVRA 244
                        :  . .                 .::                  :    * *

AmiPB1               DSSNGYTIDFNVYIGKVAGQ-------------DVSANGLGYDVVMKLMN-PYFHQGY-- 333
AmiPB2               DSITHFVSRFQVYTGRPRG--------------GQ-EHGLGERVVTELSS-DLEDGYY-- 303
AmiPB3               DASNGFVINHEVYLGKQRG--------------RVLANGLGYSVVMELMN-PFLNKNH-- 308
AmiPB4               ET-TGYLRNSYVYLGKEP-------DAIATAMEMVSRLGKSGAVIPRLME-GLLGFG--- 335
AmiPB5               ESSSGYGLVSKVYTGKENG---------------VVQTDLGRRAVMTLME-SFLDKGY-- 315
AmiPB6               DSTNGYLCDFDIYTG-KTQ--------------QGVQHGLDYSVVTKLCE-TIKGHWY-- 298
NvePB1               DANNGYVCNFKVYLG-QEN--------------RQRVHGLGYDVVMDMAK-PFLNSYR-- 308
```

```
NvePB2               DSTNCYTYDFDVYAGRQTAN-------------APSTNGLAYDVVMKLLS-PLLNQGY-- 218
NvePB3               DSESKYVVYRQQLYIGKNPG--------------ERAEVGLAARVVKELCS-GLEGMGH-- 382
TruPGBD2             DSSCGYTLNFFVDAGKDP---------------EPTGKGRSYDIVMRILNVPFLGKGY-- 364
HsaPGBD4             ESQSGYVWNALVHTGPG------------MNLKDSADGLKSSRIVLTLVN-DLLGQGY-- 326
AgaPB8               SS-EGFTWASKIYSGKSSE--------------GIRETGLAHVCIKLAEK--LFEKGR-- 322
TniPB                DSGTKYMINGMPYLGRGTQ---------------TNGVPLGEYYVKELSK-PVHGSCR-- 341
Pigibaku1            DATSSYAWNLQVYTGKPDGG-------------APEKNPRNESCPRHVSG----TQWT-- 335
BmoPB1               ESRTGYLWQMEVYTGKGHAHVVQDGEPEERGQESDEPESATAQIVLNLTR-PLFDKGH-- 221
XtrKob_DB_620        ESKTGYTGNFMIYEGT---------DSFLDPPGCPLDFTFSDKIVWELLT-PLLGRGY-- 358
XtrUri1_PCR          ESSTGYTSYFMLYEGK---------DTNLDPPGCPTDLTTSGKIVWELIT-PLLGRGY-- 344
XtrUri2_DB_469b      ESSSGYTSYFLIYEGK---------DSKLDPPGCPPDLTVSGKIVWELIS-PLLGQGF-- 338
HsaPGBD1             TT-QGYLVWFEPYQEESTMKVD-----------EDPDLGLGGNLVMNFADVLLERGQYPY 332
CinPB2               DS-SGFMYDFYIYGGKDSKQ--------LALSDDATHLQKSAQTVVKLCQHLPVKQNH-- 293
                      :   :

AmiPB1               HLYVDNFYTSVTLFKDLFAM--GVGATGTIRECRRDFPENLKDSE-VWAKGKARGSIRWG 390
AmiPB2               HLYFDNFFSTFRLMKSLLEK--GIYATATTRPNRKGFPAPLKN------AKLSRGESLVM 355
AmiPB3               HVYFDNFFSSPKLLEDLQNE--GTYACSTVRAGRVGLPPSSRR------KLKREGEMICE 360
AmiPB4               -------TLAGNLFFPLYEN--ETVACGTARKNRLMLPASMMTP---RLAK---VEHVFR 380
AmiPB5               VLYMDNYYTSVPLFEDLERR--GTLACGTVRSNRKGLPKDITDPKNAEVKALERGDSLYR 373
AmiPB6               AIFCDNFFTSYKLIEDLYRS--KILCCGTLRSGHKEFPPCLFDKD--RIKSMKRGDVFWQ 354
NvePB1               HIFCDNFFTTVRLFDHLLDQ--KTYACGTVRVNRRELPPCAKE------KLK-PGEMLQR 359
NvePB2               HLYCDNFYTSVTLIKDLFLV--KTPATGTATENRRGFPEEMKNGR-KWAGKQERGSMRWH 275
NvePB3               HLYTDNYYTSVELYQFLFDN--QIYACGTIKGNRKNFAKEVLFEN---TRGLARGSSKWL 437
TruPGBD2             KLYMDNFYTSPTLFRDLLSR--KIWACGTLRPNMAGYPK--KKGN-DMTDKTPRGTIRWM 419
HsaPGBD4             CVFLDNFNISPMLFRELHQN--RTDAVGTARLNRKQIPNDLKKR---IAK----GTTVAR 377
AgaPB8               TLYVDNLYTSYELALTCLDR--KTHLVGTLRHNKKSMPRDVLDCK------LKKGEMIX- 373
TniPB                NITCDNWFTSIPLAKNLLQEPYKLTIVGTVRSNKREIPEVLKNSR-----SRPVGTSMFC 396
Pigibaku1            QHHMRHFFTSHKLGQELLKR--KLTIVGTIRKNRSELPPQLLTSK-----NRPVKSSQFA 388
BmoPB1               TLIMDNFYNAPLLSRILKVQH-KTDSMGTLRLNREFVPEALKKK---TKKNMKEGEVAFS 277
XtrKob_DB_620        HLYVDDFYTSIPLFRALHSL--DTPACGIVSHNRKGLPRALINK---ILRH---GETYAL 410
XtrUri1_PCR          HLYVDNFYTGIPLFRALNSL--DTPACGTVHRNRKGLPRELLDK---KLKR---GEVHAL 396
XtrUri2_DB_469b      HLYVDNFYSSIPLFTALYCL--DTPACGTINRNRKGLPRALLDK---KLNR---GETYAL 390
HsaPGBD1             HLCFDSFFTSVKLLSALKKK--GVRATGTIRENRTEKCPLMNVEH-MKKMKRGYFDFRIE 389
CinPB2               QLFFDNWFTTLDLLIYLKEI--GINACGTIRGN-----EDLKKNG------RGAVDFRSD 340
                                 *              .

AmiPB1               RDPPCLALQWLDN--KVVSMLTTIDN-----ANVKKQVTRNMKNPGGGWTNNNVPQPGVI 443
```

```
AmiPB2               QKSGITACSWQDK--KKVNFFTTN--------CQPNGQGTVQRRS-RDGTLTDVNAPPCV 404
AmiPB3               QKGNLVYTKWHDK--RDVNILSTN--------FDPLEPKTVKERWKKNGDVVLVEKPACV 410
AmiPB4               RKDDMLAVRLNDK--KEIYFLTTMH------------KANVADTGKRDGCGNNVRKLQLV 426
AmiPB5               QKGTVTCVGWKDS--KMVYMLATTPV-----DPTLNSEVERSVKVDNKWQKKVVNQPSVI 426
AmiPB6               MKGPVFAITWMDK--KAVHAAGTYT-------QAPQENLPEVNRKQKDGTLQRITYPQLI 405
NvePB1               QRGQLLFTKWHDK--RDVSLLSTN--------VSPLEPGRTVRRKYRSREEIDIEKPKVT 409
NvePB2               RDGVCLAQQWKDN--RPVTILSSIEC-----ANDYVIVSRKVKS-REQWTNIEVRQPMAI 327
NvePB3               MCGPLLAVGWLDN--KAVYFLSTIHPPEFPVRAPVHARNVKRRGAGEGGQSGDIPCPPLL 495
TruPGBD2             RQGELLFVKWLDA--RQVNMCSTMHK-----AYSNDTAMRKVKNAKGEWTVKRVPIPGCI 472
HsaPGBD4             FCGELMALKWCDG--KEVTMLSTFH------------NDTVIEVNNRNGKKTKR--PRVI 421
AgaPB8               --VGIVVLKWKDS--RDVRMLSTKHAP----------YMVQTTKKCYSNQPATKLKPLTV 419
TniPB                FDGPLTLVSYKPKPAKMVYLLSSCD--------------------EDASINESTGKPQMV 436
Pigibaku1            YTADTSLVSYVPKKGKNVVLMSTLHR-------------------DGRMCDQEHHKPEII 429
BmoPB1               TTKDLSVVVWMDK--NIVAMISSFH------------PIEVGGIEK-YGYYRYK--PQVV 320
XtrKob_DB_620        RSNDLLAIKYCDT--KNEYILTTIH------------DESVIVE-HKTTNGPPKSKPLCS 455
XtrUri1_PCR          RSNELLAIKYSDT--KVVLMLTTIH------------DETMIVQ-HRSGGRPSKTKPLCS 441
XtrUri2_DB_469b      RKNELLAIKFFDK--KNVFMLTSIH------------DESVIRE-QRVG-RPPKNKPLCS 434
HsaPGBD1             ENNEIILCRWYGD--GIISLCSNAVG--------IEPVNEVSCCDADNEEIPQISQPSIV 439
CinPB2               FNSGILVTKWYDN--NAVHIGSNFVG--------IEPMSTIDRWSSEIKEKVPIQCPQII 390
                                           .

AmiPB1               ANYNKYMNAVDRSDQILATNNIHR-KCVRWWKTLFFHLID-IAIVNSYILFQEHRANSP- 500
AmiPB2               SAYNKFMGGVDYADQKRGDYKIPI-KSRRWYRYLAIFFIE-TAAVNAFILR---QLSPN- 458
AmiPB3               DLYNTSMGGVDRTDQLRSYYSACR-PSKKWYKYLFWFIFD-VSLVNSFII--FKENVDR- 465
AmiPB4               HDYNK-MGGVEHNDELLRTYYCVR-KSMKWIKKVAFHFIE-KSLLNPHNLYKK--SGGRK 481
AmiPB5               YSYNQNMGGVDLSDQRVTTYSRLM-KGSVWCLKIFFYLLE-LSMSNAQIIMAKSFGNDAP 484
AmiPB6               SSYNTYMAGVDKNDQMKSYYTIPV-SGKKWWSRILFDLVD-RSIFNSFVLE---QESPN- 459
NvePB1               DVYTAHMGGVDRADQLRSFYSSGW-QSRKWYKYMFWFLFN-LAVTNAFVLEGFKRQLQG- 466
NvePB2               NNYNSYMNGVDRSDQLMTKNNALS-KCRRWWKVLFFHMID-IAVVNSYILFQLHRAQNP- 384
NvePB3               KDYNAYMGGVDQSDQMLRYYTCIR-KTVKWYRRVLFHEVE-VAIHNAFVLECEDREGTPR 553
TruPGBD2             KDYNQHMGGVDLSDALISYYNVLH-KTLKWYKTLFYHFVD-IATVNAFILHKEMCKLQDR 530
HsaPGBD4             VDYNENMGAVDSADQMLTSYPSERKRHKVWYKKFFHHLLH-ITVLNSYILFKKDNPEHTM 480
AgaPB8               VDYNKGKCGIDYSDQMVSYATTMR-EGVKWYRKLGIELLLGTSVVNALVVFKTATKRNIG 478
TniPB                MYYNQTKGGVDTLDQMCSVMTCSR-KTNRWPMALLYGMIN-IACINSFIIYSHNVSSKGE 494
Pigibaku1            MDYNATKGGVDNMDKLVTAYSCKR-RTLRWPLVIFFDMLD-ISAYNAFVIWMALNPEWKR 487
BmoPB1               LDYNFSMGGIDHKDQMLSAYPIERSRNIIWYKKLFRRLLN-VSMHNALVMFN-HNRTHAL 378
XtrKob_DB_620        KEYSRHIDGVNKTDQIQ-YYNAAR-KTKAWYKKAAFYMIQ-MALNNSYVVYKATVPGPKL 512
XtrUri1_PCR          KEYSKHMGGVDKSDQIQHYYNATR-KTRAWYKKAAIYMIQ-MALYNSYVVYKAAVPGPRL 499
```

```
XtrUri2_DB_469b      KEYSKYMGGVDRTDQLQHYYNATR-KTRAWYKKVGIYLIQ-MALRNSYIVYKAAVPGPKL 492
HsaPGBD1             KVYDECKEGVAKMDQIISKYRVRI-RSKKWYSILVSYMID-VAMNNAWQLHRACNPGASL 497
CinPB2               TMYNKGMGGVDLADMLISLYRTEV-KTRRWYIKIFWHCID-IAKVNAWLLYRRHCNEMSI 448
                       *     .:   *               *        .   :  *.  :

AmiPB1               ---DEPALKRPASYSLTNF----REEVVNGLCGF--AEYGPPPPGHVASNLAPPSSEPSQ 551
AmiPB2               ----------HARTTQLNF----RLELINNLLGN--YCSR----------KRSAGVMEVA 492
AmiPB3               ----------RGRRTLVNF----RLALATQLIAG--FSSRSENRKRTMKAASIEATTTPE 509
AmiPB4               --------------PLLKF----KLECISALLAA----SSTDTMAPDGSN-----QFSGQ 514
AmiPB5               --------------AMLEF----RRNVIKELVNG-------KTFRLTEGNPVPAAPIPQF 519
AmiPB6               ----------HAKRTLKSF----RIDLAKQLIGD--FSSQRKRGRP--SDEPLCLRNVER 501
NvePB1               ----------KAKRTVIVF----KTELAQSLIAK--FNSR--------KRPRVPAPPTPQ 502
NvePB2               ---DNTALYRPKKYSVAEF----REELVRQLVGL--EEFGPPP------AHKPPTRAPNQ 429
NvePB3               -----------PIRTSLEF----REELAENLIGN--MRVAHNAAQRPPRNEELRLTDVGL 596
TruPGBD2             PELKQKAFREQLILSLAGIGSTPRRSAPQNHPGL--AVAAPAAP---SSSQVPDTTPPTA 585
HsaPGBD4             --------------SHINF----RLALIERMLEKHHKPGQQHLRGRPCSDDVTPLRLSGR 522
AgaPB8               ---------------IREF----REMIASELLQLP--------------VKNTTKXSATR 505
TniPB                -----------KVQSRKKFMRNLYMSLTSSFMRKR---LEAPTLKRYLRDNISNILPNEV 540
Pigibaku1            --------------VKLQKRRLFLEDLGKALVRPQIERRKHIPRTPASAAMVRRIQKENA 533
BmoPB1               --------------RHREF----RLQLAKQLLQSSRSAAPSRS-LAPAARPEPAACIRDM 419
XtrKob_DB_620        --------------SFYNY----QVQLLPSLLFG----DVEE--APDMSDNDNVVRMVGK 548
XtrUri1_PCR          --------------SYYNY----LLQLLPALLFG----DVEE--VPDMPGNDNVARMVGK 535
XtrUri2_DB_469b      --------------SYYKY----QLQILPALLFG----GVEEQTVPEMPPSDNVARLIGK 530
HsaPGBD1             ------------------------------------------------------DPLDFR 503
CinPB2               S--------KKSQRTLLNF----VVDISDNLLYRN-SCSTPTRGRPSNKRKLENDTLPGK 495


AmiPB1               FVTEHIPCFSEEKRTCVVCWQKEKKQYKVKTYCKA-------P------QCNRYMHITNE 598
AmiPB2               DGKTHFPVKGPIN----RCMQCAKTG------QRH-------RSSWRCALCDVTLCVG-C 534
AmiPB3               NAPGHFIIRREGNARKRHCVQCKKDGIKTPSNRAK-------ETIYECAQCGIALCKDPC 562
AmiPB4               HFPELIPPTPKKQTPQKKCQVCSKEGRRKQSR-----------YQCGQCNTQPGLCAAPC 563
AmiPB5               RFNREYFHYPIANNSRLACKVHIQRVDTN----------------YSCAVCGVSMCPHPC 563
AmiPB6               HFPDYLPTNESGKRKERRCKVCFNSG------VRK-------MTSYFFPDCDVGLCATPC 548
NvePB1               NIPASAHVATHVEGRKRACVLCSKKGKTTPKGYKI-------ETYYECAVCKVGLCRKGC 555
NvePB2               FETVHMPMMSDVKRNCKVCYATTGKELKVRTYCEA-------P------QCQAYLHLTST 476
NvePB3               HLPEMRQDKGNCAVCSKKIRKTHSDRYKDVPLARRPKVPYVPRPSIYCNVCNVFLCLQKD 656
TruPGBD2             PASSHLPAYFVEHMNDVTPRFRATLGRRTCVLCKR-------KSPVYCSTCKKTLCFTSL 638
HsaPGBD4             HFPKSIPATSGKQNPTGRCKICCSQYDKDGKKIRK-------ETRYFCAECDVPLCVVPC 575
```

```
AgaPB8              KSCFSHPSRFQWEKMRRACKRCYNNDRQSMGRSKVRETVKRSFTNCPSCPDQPQLCDECF 565
TniPB               PGTSDDSTEEPVMKKRTYCTYCPSKIRRKAN--------------ASCKKCKKVICREHN 586
Pigibaku1           GALSTQPTEPQSAEPEVNV-XVVNSSNKKKRCEVCG-PKMDRKTQYTCIKCKKYICNTHT 591
BmoPB1              HLP-------GKNTKKQRCKLCYSAKVQR--------------STVWRCTLQCHLCIV-- 456
XtrKob_DB_620       HFIDNIPPTLNKKYAQRACKVCRKKGIRRDVR-----------YYCPKCPSKPALCFQPC 597
XtrUri1_PCR         HFIAQIPPTPNKRYAQRKCKVCRSKGVRRDVR-----------YYCPKCPSKPALCFHPC 584
XtrUri2_DB_469b     HFIDTLPPTPGKQRPQKGCKVCRKRGIRRDTR-----------YYCPKCPRNPGLCFKPC 579
HsaPGBD1            RFVAHFYLEHNAHLSD-------------------------------------------- 519
CinPB2              KPFTPTPNEILRFDQISHWPEPRSQRGRCRLCKSG-------YSQIYCSKCHVCLCLKSS 548


AmiPB1              KIV------------------------------------ 601
AmiPB2              FERFHSGQH------------------------------ 543
AmiPB3              FLRFHSL-------------------------------- 569
AmiPB4              FKIYHTQ-------------------------------- 570
AmiPB5              FYRYHTMVDYLFNDESRNGPRRLSEGRGRPRQLRRSTEH 602
AmiPB6              FRTYHQ--------------------------------- 554
NvePB1              HQAFHEMDHGTA--------------------------- 567
NvePB2              QNCFKIWHSKEFHE------------------------- 490
NvePB3              QNCWKDFHTKVEYWR------------------------ 671
TruPGBD2            RNCYRDWHRSNSICT------------------------ 653
HsaPGBD4            FEIYHTKKNY----------------------------- 585
AgaPB8              KIVHQK--------------------------------- 571
TniPB               IDMCQSCF------------------------------- 594
Pigibaku1           VKLWPSCVV------------------------------ 600
BmoPB1              ---------------------------------------
XtrKob_DB_620       FEIYHTVVHYEKA-------------------------- 610
XtrUri1_PCR         FEVYHTVVH------------------------------ 593
XtrUri2_DB_469b     FEIYHTQLHY----------------------------- 589
HsaPGBD1            ---------------------------------------
CinPB2              NNCFKEFHCKN---------------------------- 559
```
